# Identification of cancer-driver genes in focal genomic alterations from whole genome sequencing data

**DOI:** 10.1038/srep25582

**Published:** 2016-05-09

**Authors:** Ho Jang, Youngmi Hur, Hyunju Lee

**Affiliations:** 1 Gwangju Institute of Science and Technology, School of Electrical Engineering and Computer Science, Gwangju, 500-712, South Korea; 2 Yonsei University, Department of Mathematics, Seoul, 120-749, South Korea

## Abstract

DNA copy number alterations (CNAs) are the main genomic events that occur during the initiation and development of cancer. Distinguishing driver aberrant regions from passenger regions, which might contain candidate target genes for cancer therapies, is an important issue. Several methods for identifying cancer-driver genes from multiple cancer patients have been developed for single nucleotide polymorphism (SNP) arrays. However, for NGS data, methods for the SNP array cannot be directly applied because of different characteristics of NGS such as higher resolutions of data without predefined probes and incorrectly mapped reads to reference genomes. In this study, we developed a wavelet-based method for identification of focal genomic alterations for sequencing data (WIFA-Seq). We applied WIFA-Seq to whole genome sequencing data from glioblastoma multiforme, ovarian serous cystadenocarcinoma and lung adenocarcinoma, and identified focal genomic alterations, which contain candidate cancer-related genes as well as previously known cancer-driver genes.

DNA copy number alterations (CNAs) have been studied as important genomic events involved in the initiation, development, and progression of cancer, and reported in most types of cancer^1^,^2^,^3^,^4^,^5^. Analysis of multiple cancer samples in various types of cancer has revealed that the patterns of CNAs differ depending on the types of cancer, such as deletions in chr 10 and amplifications in chr 7 in glioblastoma multiforme (GBM) and 1q amplifications in breast cancer^6^. Although such general patterns have been observed, aberrant genes may vary depending on patients with the same cancer. This might be either because pathways related to a particular cancer might be disturbed by different combinations of genes for each patient or because some genomic aberrations are random events that co-occur with driver genes^7^. Thus, it is important to distinguish driver alterations from passenger alterations. To solve this issue, previous approaches attempted to locate recurrently aberrant genomic regions in multiple patients. However, because many passenger alterations are often located in broadly aberrant recurrent regions, several algorithms such as GISTIC (Genomic Identification of Significant Targets in Cancer)^7^ and WIFA (Wavelet-based Identification of Focal genomic Alterations)^8^ considered amplitude of alterations as well as the recurrence^7^,^8^,^9^,^10^,^11^,^12^,^13^,^14^,^15^,^16^. In these algorithms, recurrent focal alterations in a relatively narrow region were distinguished from broad alterations because focal regions are more likely to contain driver alterations.

For integrating multiple samples, some algorithms require segmentation results from individual patients while others directly use raw copy number log2 ratios as the inputs^17^. Because GISTIC employed the former approach, potential signals might be lost during segmentation, and focal aberrations from a small number of samples might be buried by other samples with no change. To address such problems, WIFA and ADMIRE (Analytical Multi-scale Identification of Recurring Events) exploited the latter approach, where measurement noises were removed and then focal aberrant regions were identified after log2 ratios from single samples were summed^8^,^16^.

DNA copy numbers have been typically measured using comparative genomic hybridization arrays or single nucleotide polymorphism (SNP) microarrays. Due to the recent advance in next-generation sequencing (NGS) technologies, CNAs can be more precisely detected from sequencing data. By exploiting CNAs from NGS data, there is a chance that novel driver alterations can be discovered. Although several computational algorithms for detecting CNAs from an individual sequencing sample have been proposed^18^,^19^,^20^, accurate identification of CNAs from NGS data is a still incomplete task. It was recently reported^21^ that in a large fraction of whole genome sequencing (WGS) data for GBM samples from The Cancer Genome Atlas (TCGA)^22^, genomes consist of fractured regions with excessive read-depth changes and these regions in the WGS data do not seem to be replicated in the matched SNP array data. Many segmentation algorithms have falsely predicted CNAs due to these fractured regions. Combined with the difficulty in the individual data, identification of driver alteration regions from multiple samples is even more challenging. Most of the conventional algorithms for detecting focal genomic alterations based on array platform control false discoveries by permuting probes or segmentation results. However, in the case of NGS data, the resolution is too high to permute them.

We previously developed a WIFA method^8^ for the SNP array, which is a focal copy number alteration detection algorithm based on a wavelet transform. Wavelet transforms have been used in various applications, especially for removing noise and recovering original signals. WIFA takes the log2 ratios of SNP arrays instead of segmented results of individual samples and measures the differences between alterations in neighboring wavelet coefficients obtained by the wavelet transform. WIFA removes noise by thresholding the coefficients and locates focal regions by clustering altered regions in multiple samples. Because WIFA uses approximation of the local (high-frequency) behavior of the genomic data, it can distinguish focal aberrant regions from broad aberrations.

In this study, we developed a wavelet-based method for sequencing data, referred to as WIFA-Seq. Because NGS data have a higher resolution than SNP data and do not have predefined probes, it is challenging to test the statistical significance. In some regions, reads aligned to the reference genome are very sparse, and as a result, spurious CNAs may be detected. In addition, some NGS data have excessive read-depth changes compared to copy number changes in SNP array data. We addressed these issues in WIFA-Seq by improving WIFA. When we applied WIFA-Seq to whole-genome sequencing (WGS) data for GBM, ovarian serous cystadenocarcinoma (OV) and lung adenocarcinoma (LUAD) obtained from TCGA^22^,^23^,^24^, we found several well-known focal alterations as well as novel alterations. In addition, we compared CNA regions from WGS using WIFA-Seq with those from SNP array data using GISTIC 2.0^14^, and identified common and distinct regions.

## Materials and Methods

### Overview of the WIFA-Seq procedure

**Figure 1:**
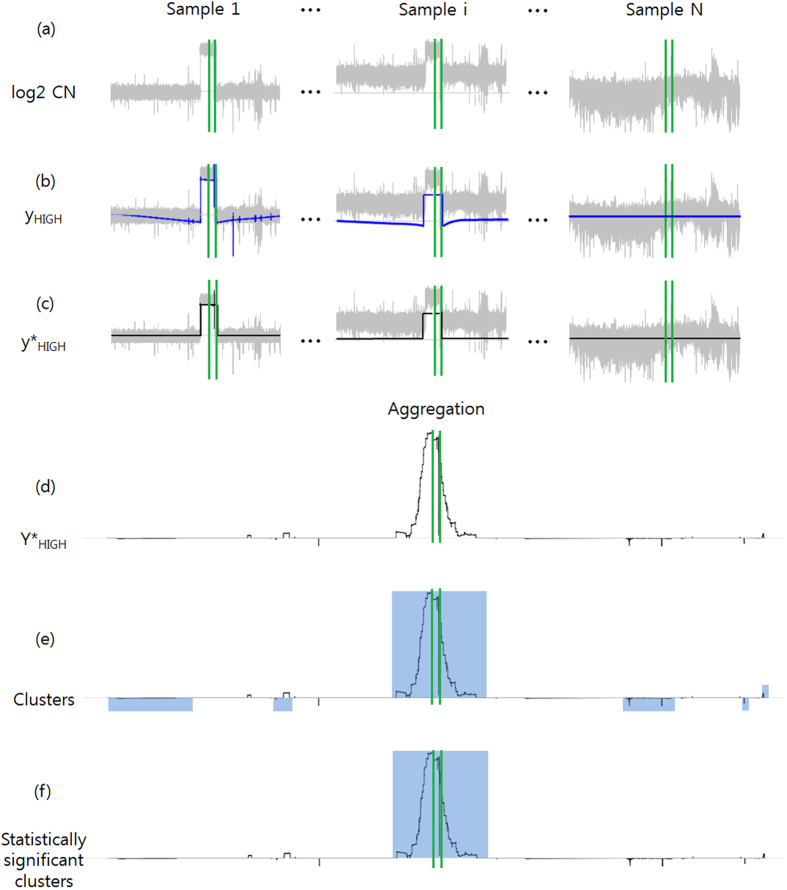
Overview of the WIFA-Seq procedure. The figure shows the detection of the recurrently altered region around EGFR indicated by the green vertical bars. (**a**) The log2 ratios of the copy number values with noise (shown in gray) from the tumor and normal WGS data were transformed into (**b**) *y*_*HIGH*_ signals (in blue) based on the wavelet coefficients, which represent copy number differences with neighbors. (**c**) After parts of signals that appear during the wavelet transform were removed, 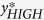 signals were obtained (in black). (**d**) All of the 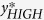 signals of individual samples were summed to a single signal called 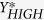. (**e**) Genomic regions were divided according to 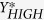 values with the same sign, and these regions are called clusters (blue rectangles). (**f**) Only clusters with statistical significance are suggested as recurrently altered regions.

To measure somatic CNAs from DNA sequencing data, tumor and normal blood samples were used. Because the total numbers of reads in the tumor sequencing data and normal sequencing data were differed, the depth of coverage (DOC) of each patient was normalized, and then the log2 ratio values between cancer and normal samples were calculated. Figure 1 illustrates the procedure of the WIFA-Seq method. First, log2 ratio values for an individual patient were converted into signals called *y*_*HIGH*_ that measure differences in the CNA values of neighboring regions. By applying a simple post-processing step to *y*_*HIGH*_ for removing artificial signals obtained during wavelet transform, 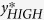 is generated. Note that because *y*_*HIGH*_ signals represent local behavior of the data, it can detect focal aberrant regions among broad aberrant regions (e.g. Sample *i* in Fig. 1). Second, individual 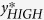 values from all patients in the same genomic locations were summed into a single value, called 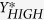, representing the extent of CNAs across multiple patients. Third, the genomes were divided into a smaller number of regions comprised of non-zero values of 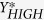, which were called clusters. Finally, the statistical significance of each cluster was determined using several false discovery control (FDC) options, and only statistically significant clusters were suggested as focal regions.

### Data sets

We obtained WGS data for patients with GBM, OV and LUAD from TCGA after acquiring authorization from the database of Genotypes and Phenotypes (dbGaP). For GBM, 37 cancer and normal paired samples were collected. The average genomic coverage of reads for somatic chromosomes was 49.6 for the cancer BAM files and 43.7 for the normal BAM files. For OV, a set of 47 cancer and normal paired samples were collected. The average genomic coverages of tumor and normal reads were 65.2 and 39, respectively. For LUAD, a set of 28 cancer and normal paired samples in a high read coverage, and another set of 70 paired samples in a low read coverage were collected. The average genomic coverages of reads for the cancer and normal samples were 47.8 and 44 for the high read coverage set and 10.4 and 10.9 for the low read coverage set, respectively.

In addition, to assess the proposed WIFA-Seq method, we manually examined recurrently altered genes in the WGS samples used in this study with the following process. First, we collected 34 known GBM driver genes from three articles^1^,^22^,^25^, which were located in CNA regions from at least one of the three articles. The 34 genes are listed in Supplementary Table 1. Second, we applied the BIC-seq segmentation method (a bin size = 100 bp and *λ* = 2.0)^18^ to each WGS sample and then manually checked whether the 34 genes were altered in the 37 GBM samples. In the manual inspection, segmented regions with an absolute log2 ratio greater than or equal to 0.4 and the length of a segment less than or equal to 25% of the chromosome arm were considered altered regions. Although some regions containing known GBM-related genes were altered compared to their neighbors, their absolute log2 ratio values were less than 0.4. In addition, for some regions with the absolute log2 ratios greater than or equal to 0.4, the relative differences with their neighbors were not high enough to be focal regions. We excluded several known GBM genes in these regions. Finally, we obtained 18 GBM genes, MDM4, FGFR3, PDGFRA, PARK2, QKI, EGFR, CDK6, MYC, CDKN2A/B, PTEN, FGFR2, CCND1, CCND2, CDK4, MDM2, RB1, and GRB2, which were altered in more than one sample in the WGS data. We refer to these genes as silver standard for the GBM WGS data. We call them the silver standard instead of the gold standard because previous studies showed that commonly altered regions do not necessarily contain cancer driver genes^7^. Supplementary Table 1 shows the alteration status of the samples.

Using a similar process applied to GBM, we have collected candidate ovarian cancer driver genes from one article^2^. Then, for the given 47 OV samples, 16 silver standard genes (MYCL, MCL1, MECOM, TACC3, ANKRD17, TERT, ID4, SOX17, MYC, PTEN, ALG8, KRAS, RB1, METTL17, NF1, and CCNE1) were collected (Supplementary Table 2). We also collected candidate lung cancer driver genes from two articles^23^,^24^. Then, for the high read coverage set of 28 LUAD samples, 12 silver standard genes (MCL1, TERT, PDE4D, CCND3, AUTS2, MYC, PTPRD, CDKN2A, CCND1, MDM2, NKX2-1, and CCNE1) were collected (Supplementary Table 3). For the low read coverage set of 70 LUAD samples, three silver standard genes (CDK4, CDKN2A, and MYC) were collected (Supplementary Table 4).

### Data pre-processing

We downloaded BAM files of WGS from the Cancer Genomics Hub (CGHub), where reads were mapped to a human genome reference sequence. We counted the aligned read in a one-base pair bin from the BAM files and remove outliers using the method from^18^ with the sliding window of 200 bp and the quantile of 0.95. We then divided the genomic regions into consecutive 100 bp bins and recalculated read count within these bins. Next, we calculated the log_2_ ratio for the somatic copy number alteration signal as follows: 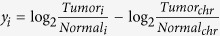, where *Tumor*_*i*_ is the number of aligned reads within 100 bp bin *i* in the tumor BAM files, *Normal*_*i*_ is the number of aligned reads within 100 bp bin *i* in the corresponding normal BAM files, and *Tumor*_*chr*_ and *Normal*_*chr*_ are the total number of aligned reads in the chromosome where the bin *i* belongs to, respectively.

### Wavelet transform for an individual patient

Wavelet transform is used to convert original log2 ratio copy number signals that are 2^*J*^ long into scaling coefficients and wavelet coefficients. We used the Haar wavelet and obtained wavelet coefficients by computing the difference between the neighboring signals and scaling coefficients by computing the average of the neighboring signals. Scaling coefficients were transformed into lower-level of wavelet coefficients and scaling coefficients. Starting from level *J*, one continues this process recursively until one reaches the level *M*. Stationary wavelet transform was used because conventional discrete wavelet transform lacks translation-invariance property. The lengths of the chromosomes are not given as a perfect power of 2, in general. To solve the problem of wavelet transform on the boundaries of the chromosome, the signals were extended by duplicating the original signals symmetrically and concatenating them. After the wavelet transform was applied to these extended signals, we can get *y*_*HIGH*_ by extracting the values within the range of the original signals.

**Figure 2:**
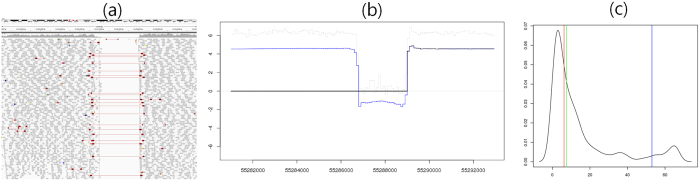
A region unmapped to the reference sequence in EGFR. (**a**) An integrative genome viewer plot of normal samples around EGFR shows a region unmapped to the reference genome. (**b**) This region can be falsely considered to have copy number changes. The gray signal represents the log2 ratios, the blue line *y*_*HIGH*_ signals, and the black line 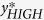 signals. (**c**) When we collected the segments with the nonzero 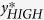 values and drew the distribution of the number of normal reads per its size, it usually showed a bimodal-like shape and the falsely identified segments were close to the left-side peak. We excluded these false segments and re-ran the wavelet procedure.

The observed copy number alteration signal *y*_*i*_ is represented as *y*_*i*_ = *f*(*x*_*i*_) + *e*_*i*_, where *x*_*i*_ is the genomic location, *e*_*i*_ is noise, and *f*(*x*_*i*_) is the true copy number change in *x*_*i*_ location. The goal of conventional wavelet denoising is to remove *e*_*i*_ and to restore *f*(*x*_*i*_). The original signal *y*_*i*_ is transformed into wavelet coefficients and scaling coefficients, the noises can be estimated with the following formula 
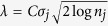
, where 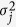 is the noise variance estimate from the wavelet coefficients in level *j*, and *n*_*j*_ is the number of coefficients in level *j*. *C* is a customized argument that a user can adjust. Hard thresholding by setting wavelet coefficients ≤*λ* as zero is used to modify the wavelet coefficients. Let us denote *s* scaling coefficients, *w*_*HIGH*_ wavelet coefficients, and 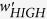 wavelet coefficients after hard thresholding. Then, the transform that produces the thresholded wavelet coefficients is represented as 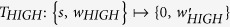. To make *y*_*HIGH*_ signals for every individual patient, we used the following modified wavelet transform and inverse-transform procedures


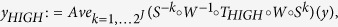


where *W* is the Haar wavelet transform, and *S* is the shifting operator. Thus, *y*_*HIGH*_ is the measure of the difference of copy numbers with their neighbors. *y*_*HIGH*_ always generates opposite-signed values next to the true difference values. Thus, regions with *y*_*HIGH*_ values whose signs are different from the log2 ratio of these regions are set to zero, and this modified signal is called 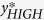. (See Fig. 2 in^8^ for details.) Figure 1(c) shows the final profiles for individual patients with GBM.

To apply the wavelet-based approach to WGS data, some genomic regions that might be attributed to false CNA regions should be removed in advance. One type of abnormal region is the ‘N’ sequence region. Because reads were not mapped to the reference sequence regions of centromeres and heterochromatins (set to ‘N’ bases), we reduced the effect of these gaps by concatenating all of the regions with non-‘N’ bases before we applied wavelet transform (Supplementary Fig. 1(a)). Another type of abnormal region is the region without aligned normal reads. There were few reference sequence regions, to which sequencing reads were mapped very sparsely. In some cases, none of reads were mapped (Fig. 2(a)). Because these genomic regions can be mistakenly considered to be copy number deletions (Fig. 2(b)), these regions should be removed. Thus, we applied the wavelet transform to find these genomic regions (Supplementary Fig. 1(b)). For a normal sample, the average number of reads mapped on these regions is usually smaller than that on other regions (Fig. 2(c)). We applied K(=2)-means clustering to these genomic segments. If the segment belonged to the class of a smaller average number of mapped reads and the length of the segment was greater than or equal to a pre-specified length (3 KB), we removed these segments. After we removed these genomic regions, we applied the wavelet transform again (Supplementary Fig. 1(d)).

### Integrating CNA regions for multiple patients


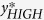
 signals from individual patients were aggregated into 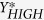 for multiple samples. 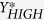 is a signal representing alterations in multiple patients by adding the 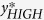 values of the same genomic location of all patients (Fig. 1(d)). Then, genomic regions were divided into sub-regions called clusters using 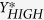 as described in^8^. Briefly, regions with continuous nonzero-values 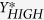 were grouped if the regions were located within the distances *d*. The regions with nonzero values in the same group were subgrouped if they are within the distance *r*. The score of each subgroup is calculated by adding the 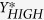 values. For each group, one subgroup with the highest absolute score was considered a focal altered region, and called a cluster. Only clusters with statistical significance are suggested as focal regions, and we made several options to determine the statistical significance of the clusters, as described in the next subsection.

### False discovery control (FDC) options

We determined the statistical significance of the clusters by calculating *p*-values using different false discovery control options. The statistical significance of the cluster was tested under the null hypothesis that genomic regions in patients are independently altered. For this task, for each patient, regions with consecutive nonzero values were randomly reallocated across the chromosome. Then, random clusters from multiple patients were generated by 1,000 independent random allocations. To determine the statistical significance of the observed clusters, the FDCs were measured in five different ways. In FDC1, a score of the observed cluster was compared to the maximum score of random clusters in the *i*-th permutation 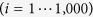. The number of cases that the maximum score of each permutation was greater than the observed cluster score was counted and the *p*-value was calculated by dividing it by 1,000. In FDC2, the score of the observed cluster was compared to the maximum score of random clusters, the length of which was less than or equal to the length of the observed cluster. In FDC3, the score of the observed cluster was compared to the maximum score of random clusters, where the length of random clusters was less than or equal to the length of the observed cluster, and the number of patients with alterations in the random cluster was less than or equal to the number of patients with alterations in the observed clusters. In FDC4, linear regression coefficients were estimated from the relationship between the lengths of the random clusters and their scores, and the score of the observed cluster was compared to the randomly estimated score from the linear regression function. In FDC5, another variable representing the number of patients in the observed cluster was added for the estimation of the linear regression coefficients.

### Parameters in WIFA-Seq

WIFA-Seq has five parameters: *J*, *M*, *C*, *d* and *r*. *J* represents the exponent of the binary logarithm of the signal length to be analyzed. To analyze about 3.2 billion base (MB) pairs of human genomes, we divided the genome into consecutive 32,000,000 bins, where the size of a bin was 100 base pairs. Thus, *J* was fixed as 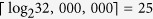. Because WIFA-Seq measures the difference between neighboring signals, we needed to decide the length of genomic regions for the differences with neighbors. For example, to incorporate the difference of neighbors up to 3 MB, which is 30,000 bins, wavelet transform was applied up to 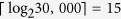 levels. In this case, *M* was set as 25 − 15 = 10. *C* controlled the degree of thresholding and was adjustable depending on the noise level in the data. *d* and *r* controlled the size of the candidate focal regions called clusters. After trying different values for *M*, *C*, *d* and *r* using the GBM data set, we fixed the parameters at *M* = 10, *C* = 2.0, *d* = 0.3 MB and *r* = 0.3 MB. To show that these parameter values can be used for other data sets, we used the same values for the OV and LUAD data sets. These parameters were used in the WIFA method^8^ as well, and more details about the parameters can be found there.

## Results

### The number of segments in 37 patients with GBM

**Figure 3:**
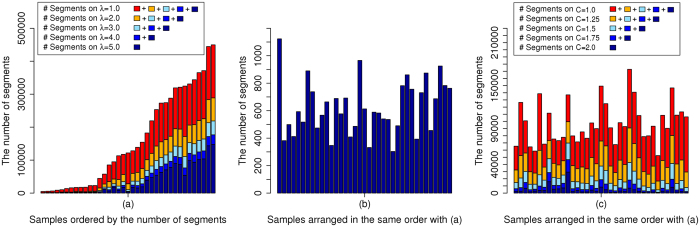
The number of segments from depth coverage of the sequencing data. (**a**) The numbers of segments from WGS data for 37 GBM samples are shown when BIC-seq was applied with a parameter *λ* ranging from 1.0 to 5.0. Each bar represents the number of segments of an individual sample and samples are ordered by the numbers of segments with *λ* = 1.0. Because the numbers of segments increase when values for *λ* decrease, bars are segmented with the numbers of segments with *λ* = 5.0 (dark blue) and the increased numbers of segments with the changes of *λ* from 5.0 to 4.0 (blue), from 4.0 to 3.0 (sky-blue), from 3.0 to 2.0 (orange), and from 2.0 to 1.0 (red). (**b**) The numbers of segments of level 3 SNP array data from TCGA for the same samples are shown, sorted in the same order as (**a**). (**c**) The numbers of segments for the same samples are shown when WIFA-Seq was applied to WGS data. When the noise threshold *C* increases, the numbers of segments for all the samples dramatically decreased.

Because a subset of TCGA GBM samples has fractured regions with excessive read-depth changes^21^, we examined whether WIFA-Seq contolled these excessive changes by comparing them with BIC-seq^18^ and TCGA SNP array data^22^. When we applied BIC-seq to WGS data for 37 GBM samples with a parameter *λ* ranging from 1.0 to 5.0, the total numbers of segments significantly vary among samples (1,000 ~ 500,000) (Fig. 3(a)). On the other hand, for matched level 3 SNP array data from TCGA, most of the samples have less than one thousand segments and the difference of the number of segments among samples was less than four times (Fig. 3(b)). Figure 3(c) shows the number of segments from 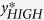 signals with parameters of *J* = 25, *M* = 10 and *C* ranging from 1.0 to 2.0. Note that although WIFA-Seq is not a segmentation algorithm for a single sample, we here define a segment in 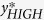 signals as a consecutive genomic region with the same sign for 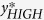 values, only to check whether WIFA-Seq contolled these excessive read-depth changes in the NGS data. Although the total numbers of 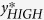 segments at the noise threshold *C* = 1.0 were high, the increase was not rapid compared to those in Fig. 3(a). When we gradually increase the threshold *C*, the numbers of segments were dramatically reduced and fluctuations of the numbers of segments among samples were not significant.

### Focal copy number alterations in 37 patients with GBM

WIFA-Seq was applied to the 37 patients with GBM. To investigate the effect of various parameters, WIFA-Seq was conducted using parameters *M* ranging from 8 to 17, *C* ranging from 0.5 to 3.0, a group distance *d* taken from a set of several different values, and a cluster distance *r* taken from another set of several different values. Table 1 shows 25 statistically significant clusters (a significant level of 0.1 in FDC1) with the parameters of *J* = 25, *M* = 10, *C* = 2.0, *d* = 0.3 MB and *r* = 0.3 MB ordered by the cluster scores. Out of 18 silver standard genes, 10 genes, including EGFR, CDKN2A/B, CDK4, MDM4, PDGFRA, MDM2, PTEN, PARK2, and QKI, were identified. These genes were located in highly ranked clusters, showing that clusters with high scores might contain cancer driver genes. All genes included in the clusters are shown in Supplementary Table 5.

**Figure 4:**
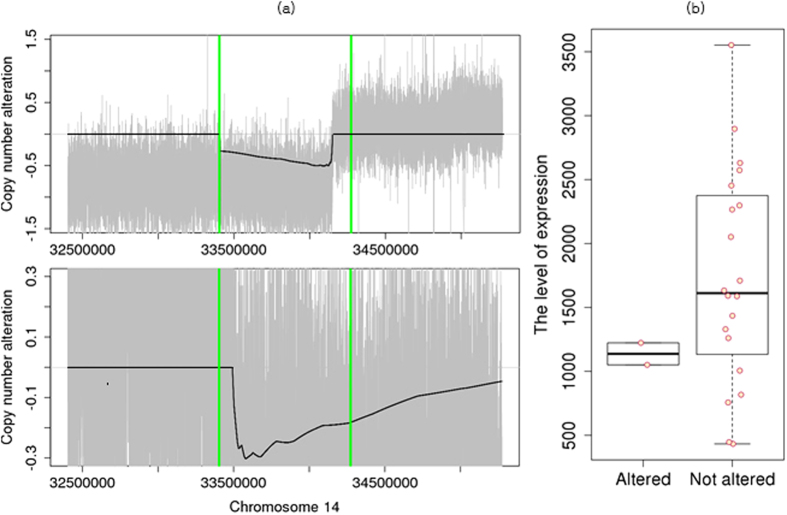
Copy number deletions in NPAS3. (**a**) Individual profiles of two samples that have copy number deletions. Gray signals represent log2 ratios of the copy numbers, and the black line represents 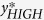 signals. NPAS3 is located between green vertical bars. (**b**) Boxplots of expression levels of samples with NPAS3 deletion and samples without copy number changes.

Although *neuronal PAS domain protein 3* (NPAS3) was not included in the silver standard, it was identified in the deleted regions of two GBM samples with statistical significance on FDC1 (Fig. 4). In astrocytomas, NPAS3 is reported as a tumor suppressor, which is involved in cell cycles, apoptosis, and cell migrations^26^. When we compared the expression levels of this gene using RNA-seq from the TCGA data, the expression levels of the two samples with deletions were lower than those of other samples (Supplementary Table 6). In Catalogue Of Somatic Mutations In Cancer (COSMIC) database^27^, we also found focal deletions harboring NPAS3 in other four TCGA GBM samples, which were not included in our GBM WES data set. It may show that deletions around NPAS3 did not occur by chance.

**Figure 5:**
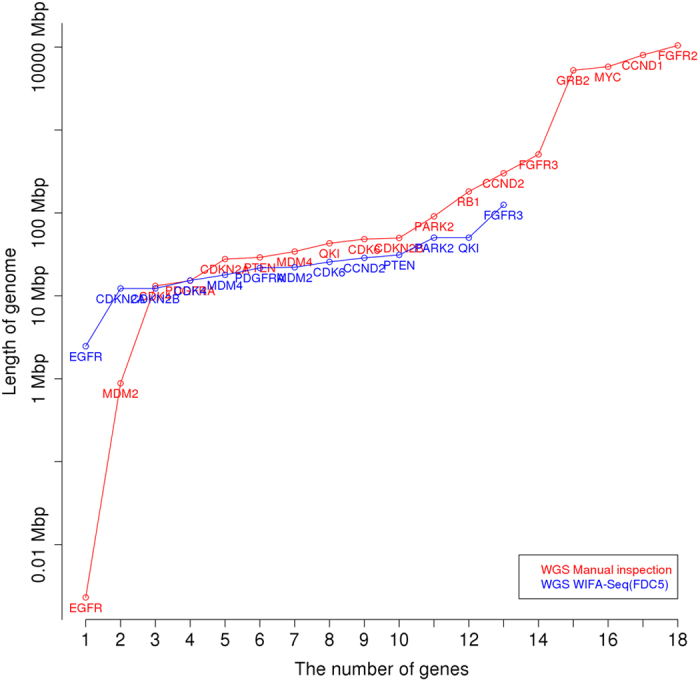
Comparison between manual inspection and WIFA-Seq for GBM. The *x*-axis represents the number of known GBM genes, and the *y*-axis represents the length of the genomes for identifying the genes. For the *y*-axis, the accumulated length of clusters sorted by their scores was used for WIFA-Seq, and the accumulated length of the segments sorted by the log2 ratio values of the segments was used for the manual inspection. The red and blue lines represent the costs of manual inspection and WIFA-Seq, respectively.

Although WIFA-Seq identified only some of the genes in the silver standard, which is obtained by a manual inspection, WIFA-Seq might suggest shorter genome regions than a manual inspection for identifying cancer driver genes. As described in the Data sets section, prior knowledge from the literature was used to find silver standard genes. If we do not have such prior knowledge about a given cancer, we needed to inspect the segmented region sorted by the log2 ratio until the genes were found. Thus, we compared the manual inspection with WIFA-Seq by comparing the length of the genomes for identifying known GBM genes in the silver standard. To measure the length, for WIFA-Seq, the accumulated length of clusters sorted by their scores was used, and for manual inspection, the accumulated length of segments sorted by the log2 ratio values of the segments was used. Figure 5 shows that the length required to discover known GBM genes with manual inspection is larger than that by WIFA-Seq, where WIFA-Seq used the FDC5 option to detect statistically significant clusters and identified 13 genes out of 18 silver standard genes. For identifying 13 genes, the manual inspection and WIFA-Seq require searching about one MB pairs and about 13 MB pairs, respectively, (Supplementary Table 7), showing that if we have no prior knowledge about these genes, the validation costs for a manual inspection dramatically increase compared to those for WIFA-Seq.

**Figure 6:**
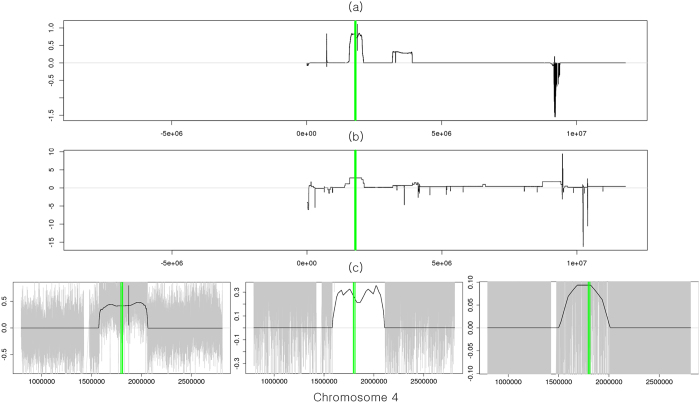
Copy number alterations in FGFR3. (**a**) Sum of the log2 ratios in the segments from the SNP array data. FGFR3 is located between green vertical bars. (**b**) The focal aberrations identified by WIFA-Seq are clearly distinguished from its neighbors in the 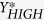 signal compared to the SNP array. (**c**) Individual profiles of three samples that have copy number amplification in FGFR3. The gray signals are log2 ratios, and the black line is 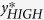.

We compared cancer driver genes identified from two platforms of the SNP array and WGS using the same 37 patients with GBM. For the SNP array, GISTIC 2.0 (a FDR *q*-value < 0.25 and a confidence level = 90) was used to identify the focal aberrant regions, and peaks whose length were greater than 25% of the arm length were regarded as broad alterations and excluded. All focal regions and genes identified by GISTIC 2.0 are shown in Supplementary Table 8. Because we used two different platforms, we chose 14 genes (MDM4, FGFR3, PDGFRA, QKI, EGFR, CDK6, CDKN2A/B, PTEN, FGFR2, CCND2, CDK4, MDM2, GRB2) as another silver standard set whose recurrent occurrence was manually confirmed from TCGA level 3 segments in the SNP array and the segments obtained by applying the BIC-seq method (a bin size = 100 bp and *λ* = 2.0)^18^ to WGS. Table 2 shows the genes identified from WIFA-Seq clusters and/or GISTIC peaks. WIFA-Seq found 12 genes out of a total of 14 genes while GISTIC 2.0 found nine genes. FGFR3 in chr 4, EGFR in chr 7, and CDKN2B in chr 9 were identified only by WIFA-Seq. Note that FGFR3, CDK6, and CCND2 could be found with the FDC4 and/or FDC5 options, which requires inspections for longer genomic regions than FDC1. For EGFR, the peak from GISTIC was too narrow so it did not intersect with EGFR. For CDKN2B, it was closely located to CDKN2A in chr 9, and CDKN2A and CDKN2B were included in the same cluster in WIFA-Seq. However, in GISTIC, only CDKN2A was included in a narrow peak. Although we confirmed significant alterations around FGFR3 in both segments from the SNP array and segments in WGS, no peaks intersected with FGFR3 in GISTIC 2.0. In WIFA-Seq, the cluster around FGFR3 was statistically significant only in FDC5. Figure 6(a) shows the sum of the segmented regions of three samples from the SNP array, and Fig. 6(b) shows the 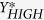 signals around the FGFR3 regions. Amplifications around FGFR3 are more clearly identifiable in the 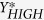 signals than in the sum of the individual segmentations. We also confirmed copy number amplifications around FGFR3 in the three GBM samples using individual 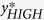 signals (Fig. 6(c)).

**Figure 7:**
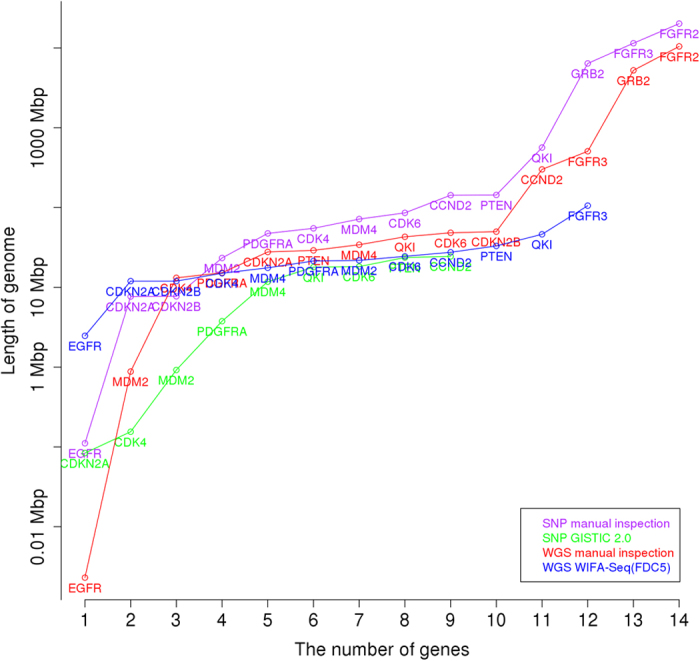
Comparison between the SNP array and WGS in GBM. The *x*-axis represents the number of known GBM silver standard genes for the SNP array and WGS, and the *y*-axis represents the length of the genomes for identifying these genes. Manual inspection and GISTIC 2.0 for the SNP array, and manual inspection and WIFA-Seq for WGS were compared.

We further compared CNA regions identified with four methods: CNAs obtained by manual inspection of the SNP array data from TCGA level 3 data, where segments were obtained with a circular binary segmentation algorithm, CNAs by applying GISTIC 2.0 to the SNP array data, CNAs by the manual inspection of the WGS data after segmentation by BIC-seq (*λ* = 2.0), and CNAs by applying WIFA-Seq to the WGS data. Even though the total inspection length by WIFA-Seq with the FDC5 option is larger than that by GISTIC 2.0, WIFA-Seq found three more genes than GISTIC 2.0 (Fig. 7). In addition, the length required to find up to 12 silver standard genes by manual inspection after the BIC-seq segmentation was about four times larger than that with WIFA-Seq (Supplementary Table 9).

### Focal alterations in 47 patients with OV

We have applied WIFA-Seq to the 47 patients with OV. Table 3 shows 34 statistically significant clusters (a significant level of 0.1 in FDC1) with the parameters of *J* = 25, *M* = 10, *C* = 2.0, *d* = 0.3 MB and *r* = 0.3 MB, ordered by the cluster scores. Out of 16 silver standard genes, ten genes, including MYCL, MECOM, TACC3, ANKRD17, MYC, PTEN, ALG8, KRAS, METTL17, and CCNE1, were identified. All genes included in the clusters are shown in Supplementary Table 10.

**Figure 8:**
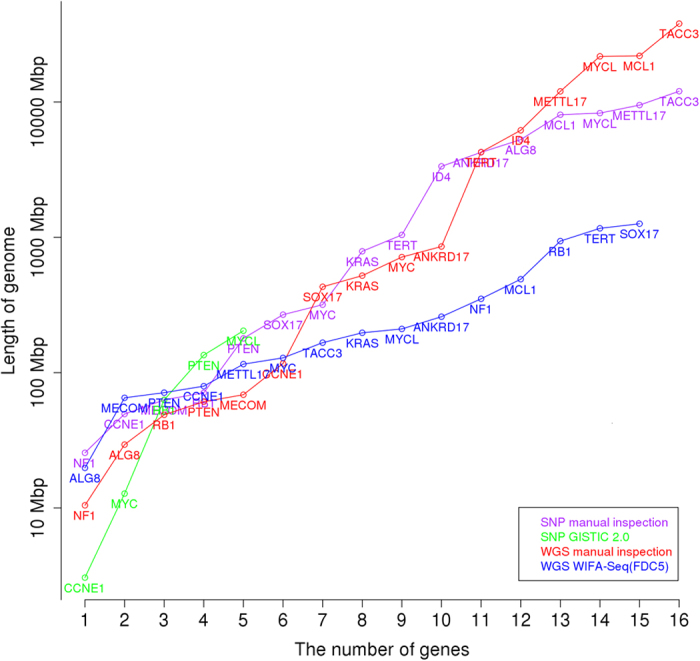
Comparison between the SNP array and WGS in OV. The *x*-axis represents the number of known OV silver standard genes for the SNP array and WGS, and the *y*-axis represents the length of the genomes for identifying these genes. Manual inspection and GISTIC 2.0 for the SNP array, and manual inspection and WIFA-Seq for WGS were compared.

For comparison with the SNP array data, GISTIC 2.0 (a FDR *q*-value < 0.25 and a confidence level = 90) was applied to the same 47 patients with OV. All focal regions and genes identified by GISTIC 2.0 are shown in Supplementary Table 11. For comparison of the CNAs identified from two different platforms, we used the 16 silver standard genes that were used in the WGS data analysis and were also identified in SNP array data by the manual inspection. Table 4 shows the genes identified from WIFA-Seq clusters and/or GISTIC peaks. WIFA-Seq found 15 genes with a significant level of 0.1 in FDC4 and FDC5 (MYCL, MCL1, MECOM, TACC3, ANKRD17, TERT, SOX17, MYC, PTEN, ALG8, KRAS, RB1, METTL17, NF1, and CCNE1) out of total 16 silver standard genes while GISTIC 2.0 found five genes (MYCL, MYC, PTEN, RB1, and CCNE1). Because the range of the peaks containing SOX17 was too broad in GISTIC 2.0 (about 61 MB), these peaks were not regarded as focal alterations. The comparisons among the manual inspection of the SNP array data and the WGS data, GISTIC 2.0, and WIFA-Seq are shown in Fig. 8 and Supplementary Table 12. WIFA-Seq located more genes than GISTIC 2.0 and the similar number of genes as the manual inspection in the shorter genomic regions.

### Focal alterations in 28 patients with LUAD

WIFA-Seq was applied to WGS data from 28 LUAD patients. We used the same WIFA-Seq parameter values (*J* = 25, *M* = 10, *C* = 2.0, *d* = 0.3 MB and *r* = 0.3 MB) as those used in the patients with GBM. Table 5 shows 23 clusters with a statistically significant level of 0.1 with the FDC1 option. When we compared the identified clusters with the 13 silver standard genes, CDKN2A and CCNE1 were identified. All genes included in the clusters are shown in Supplementary Table 13.

**Figure 9:**
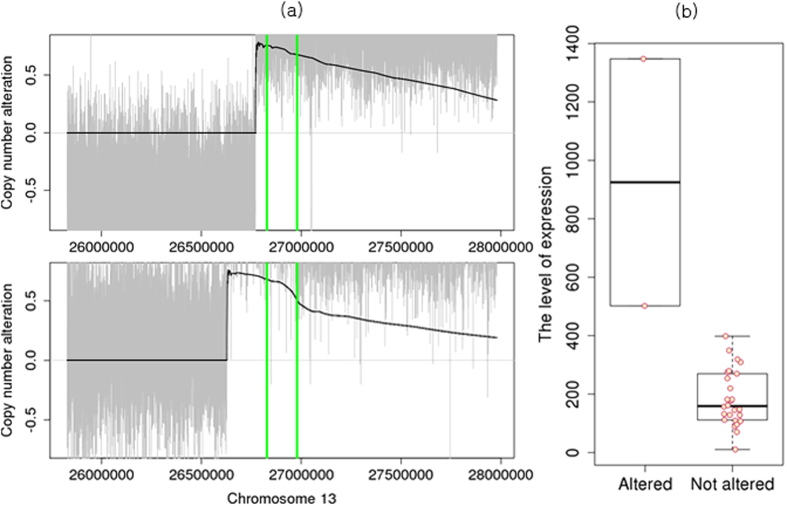
Copy number amplifications in CDK8. (**a**) Individual profiles of two samples that have copy number amplifications. The gray signals represent the log2 ratios of copy numbers and the black line represents the 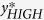 signals. CDK8 is located between green vertical bars. (**b**) Boxplots of the expression levels for samples with CDK8 amplification and samples without copy number changes.

WIFA-Seq identified amplifications of CDK8. Figure 9 shows two samples with amplifications in CDK8, and these samples have the highest expression level in CDK8 (Supplementary Table 6). Previous studies showed that in colorectal cancers, the region around CDK8 is known to be recurrently altered, and the gene is reported to be a oncogene because it modulates beta-catenin activity^28^. In addition, we observed two amplifications around ZNF521. ZNF521 is listed in the COSMIC Cancer Gene Census (CGC) database^29^, and it was reported that overexpression of ZNF521 is closely related to the growth and proliferation of medulloblastoma cells^30^.

For comparison with the SNP array data, GISTIC 2.0 (a FDR *q*-value < 0.25 and a confidence level = 90) was applied to the same 28 patients with LUAD. All focal regions and genes identified by GISTIC 2.0 are shown in Supplementary Table 14. For comparison of the CNAs identified from two different platforms, we chose six silver standard genes (TERT, PDE4D, PTPRD, CDKN2A, MDM2 and CCNE1). Table 6 shows the genes identified from WIFA-Seq clusters and/or GISTIC peaks. WIFA-Seq found four genes (TERT, PTPRD, CDKN2A and CCNE1) while GISTIC 2.0 found two genes (CDKN2A and MDM2). Because the ranges of the peaks containing PDE4D, TERT and CCNE1 were too broad in GISTIC 2.0 (more than 50 MB), these peaks were not regarded as focal alterations. However, WIFA-Seq located regions containing CCNE1 more precisely than GISTIC 2.0. The comparison between the SNP data and the WGS data using manual inspection, GISTIC 2.0, and WIFA-Seq for the LUAD data is shown in Supplementary Table 15.

In addition, we applied WIFA-Seq to WGS data with a low read coverage obtained from 70 patients with LUAD. Only three silver standard genes (CDK4, CDKN2A and MYC) were confirmed by the BIC-seq segments due to the low coverages of the data (Supplementary Table 4). When we applied WIFA-Seq with the same parameters *M* = 10 and *C* = 2.0 as those used in the GBM and the high coverage LUAD data, WIFA-Seq identified CDKN2A with the FDC1 option. WIFA-Seq also found a focal region that had HOXA9 with the FDC5 option. HOX is known to be related to the development of various human cancers, including LUAD^31^. All genes included in the clusters are shown in Supplementary Table 16. To discover more focal CNA regions, we can lower the threshold, although it might give false discoveries. Further analysis is required to increase the sensitivity for identifying focal regions when low coverage WGS data are used.

## Discussion

We applied WIFA-Seq to the WGS data to identify recurrent focal CNAs and compared it with GISTIC 2.0, which uses SNP array data. WIFA-Seq found most of the genes identified by GISTIC 2.0 as well as novel candidate oncogenes and tumor suppressor genes. Although the parameters *M*, *C*, *d* and *r* are adjustable in WIFA-Seq, we suggest default values of *M* = 10, *C* = 2.0, *d* = 0.3 MB and *r* = 0.3 MB. The value *J* = 25 was determined by the length of the genome and the resolution of the platform. We selected these values from the GBM data set after trying different values and then applied them to the OV and LUAD data sets. Using these default parametric values, we successfully recovered previously known cancer-related genes in the WGS data sets of the three different cancer types. We also suggested similar default values for the SNP array data of the previously developed WIFA method^8^. For the SNP array data, the actual length of genomes we considered to generate 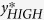 values was about 1.6 MB (*J* = 17 and *M* = 12 for GBM from the Affymetrix 100 K SNP array, and *J* = 18 and *M* = 11 for lung cancer from the 250 K Sty SNP array). For the WGS data sets, the actual length of neighboring genomes for focal aberrations was about 3.2 MB (*J* = 25 and *M* = 10 for WGS GBM, OV and LUAD data sets. See the Parameters in WIFA-Seq section for details on the calculations). When we tested 1.6 MB for the length of neighoring genomes for the WGS data sets, the identified focal regions were similar when 3.2 MB was considered. For the SNP array, *C* = 1.94 was used, which was similar to the value we used for WGS. Taken together, the threshold value of *C* of around 2.0 and the length of neighboring genomic regions from 1.6 MB to 3.2 MB demonstrate robust performances regardless of tumor types and platforms.

We also developed various methods for assigning statistical significance to the candidate focal regions. We randomly reallocated non-zero 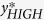 regions across the chromosome, produced random clusters, and compared the value of the observed cluster to the maximum value of the random clusters. It gave conservative *p*-values to the observed clusters (the FDC1 option). When we considered the length of focal altered regions and the number of samples (the FDC2, FDC3, FDC4, and FDC5 options), they gave less strict *p*-values, which generated more statistically significant clusters and have the potential to find novel focal altered regions.

In this study, we used only DNA copy number data to identify cancer driver genes. However, one study reported that genes with copy number amplifications and high gene expression levels are more likely to be cancer driver genes^32^. In that study, GISTIC 2.0 was applied to breast cancer cell lines and then genes within amplified peaks with a G-score > 0.3 and with expression levels that were within the top 50% of the expressed genes for the cell line were selected. Hence, we applied a similar approach of combining CNAs and gene expressions to identify cancer driver genes more accurately. In our study, we used 29 GBM samples and 47 OV samples in TCGA, which have RNA-seq, SNP array, and WES data. We first selected genes whose genomic regions were predicted as amplification or deletions by WIFA-Seq or GISTIC 2.0. Next, we selected genes with expression levels that were within the top 50% in all expressed genes of each sample (for amplified genes) or below the top 50% (for deleted genes) for at least half of the samples. Supplementary Fig. 2 shows the number of genes to be investigated for identifying cancer driver genes when WIFA-Seq (FDC5) and GISTIC 2.0 were applied with and without gene expression data. For both the WIFA-Seq (FDC5) and GISTIC 2.0 methods that were applied to the SNP array and WES data, respectively, the integration of gene expression data required fewer genes to be investigated for identifying cancer driver genes. However, some driver genes were not identified because their expression level changes were not significant. Overall, the integration of gene expression level data was helpful for discovering genomic regions related to cancer.

In this study, we evaluated the WIFA-Seq method by the number of previously known cancer driver genes the method had identified. However, because the number of known cancer type-specific genes was small compared to the number of genes included in the focal aberrant regions identified by WIFA-Seq, we performed a functional enrichment test to investigate whether the genes in the focal regions were enriched with functions related to cancer hallmarks. Among ten cancer hallmarks listed in Wang *et al*.^33^, we found that gene ontology biological process terms related to five cancer hallmarks (regulation of cell proliferation, resisting programed cell death, induction of angiogenesis, abnormal metabolic pathways, and evading the immune system) were enriched for the GBM, OV, and LUAD data sets (Supplementary Table 17). In addition, we found that many genes in the focal regions were included in the COSMIC CGC^29^ and that the numbers of genes in the CGC increased when we changed the FDC options (Supplementary Table 17), suggesting that these genes might be candidate cancer driver genes for each cancer type.

## Additional Information

**How to cite this article**: Jang, H. *et al*. Identification of cancer-driver genes in focal genomic alterations from whole genome sequencing data. *Sci. Rep*. **6**, 25582; doi: 10.1038/srep25582 (2016).

## Supplementary Material

Supplementary Information

Supplementary Tables

Supplementary Dataset 1

Supplementary Dataset 2

Supplementary Dataset 3

Supplementary Dataset 4

Supplementary Dataset 5

## Figures and Tables

**Table 1:** Statistically significant clusters (FDC1) from WIFA-Seq for 37 patients with GBM.

Chr	Start	End	Score	# of patients	*P*-values	Genes
7	53982101	56449401	504208.00	25	0.00	EGFR
9	19626001	29431601	−156930.60	26	0.00	CDKN2A|CDKN2B
12	56919201	59971201	119463.40	11	0.00	CDK4
1	203277901	205825501	110989.60	7	0.01	MDM4
4	52663201	56488401	97355.73	7	0.00	PDGFRA
12	69038101	69416901	52436.32	6	0.01	MDM2
6	106784301	108840801	49466.33	4	0.04	
10	88984001	90359501	−13045.48	14	0.06	PTEN
18	25736501	27397901	−10002.23	9	0.01	
19	34288701	39415601	9068.60	7	0.00	
12	16587901	21884101	−8619.56	8	0.07	
6	162272701	164169001	−7932.19	3	0.03	PARK2|QKI
2	233933901	235859501	−7083.54	2	0.00	
15	35156501	37062401	−6761.21	14	0.06	
11	66176001	67434201	6164.80	2	0.01	
16	4027401	5129501	6045.22	2	0.02	
16	84363801	85714601	5984.56	4	0.02	
14	33407901	35914401	−5872.19	3	0.04	NPAS3*
21	9411001	11188101	−5284.48	23	0.00	
7	153749801	156394701	−4123.94	5	0.02	
8	1902201	2776501	−3727.91	3	0.01	
4	49095601	49660001	−3547.28	4	0.02	
20	58229601	58514701	−2890.01	16	0.00	
20	3684901	4517301	2882.16	2	0.04	
8	43027601	43838801	1316.54	2	0.01	

Clusters are ordered by cluster scores. Among the genes in the clusters, some genes related to GBM are specified. Previously known GBM genes in the silver standard are shown without asterisks.

**Table 2:** Comparison between GISTIC and WIFA-Seq for GBM.

Chr	Genes	WIFA				GISTIC		
		cluster.start	cluster.end	cluster.length	FDC	peak.start	peak.end	peak.length
1	MDM4	203277901	205825501	2547601	1|2|3|4|5	204334766	204529807	195042
4	FGFR3	737301	2108301	1371001	5			
4	PDGFRA	52663201	56488401	3825201	1|2|3|4|5	55140876	55218386	77511
6	QKI	162272701	164169001	1896301	1|2|3|4|5	163767962	165698161	1930200
7	EGFR	53982101	56449401	2467301	1|2|3|4|5			
7	CDK6	91140201	92642101	1501901	4|5	92240329	92427373	187045
9	CDKN2A	19626001	29431601	9805601	1|2|3|4|5	21959052	21976869	17818
9	CDKN2B	19626001	29431601	9805601	1|2|3|4|5			
10	PTEN	88984001	90359501	1375501	1|2|3|4|5	89617158	90034038	416881
12	CCND2	3257601	4622101	1364501	4|5	4374374	4436676	62303
12	CDK4	56919201	59971201	3052001	1|2|3|4|5	58125396	58162738	37343
12	MDM2	69038101	69416901	378801	1|2|3|4|5	69178021	69260755	82735

For both methods, genes were included in clusters/peaks. Thus, start and end positions and the length of clusters/peaks are specified. A column FDC indicates FDCs that identified the given genes.

**Table 3:** Statistically significant clusters (FDC1) from WIFA-Seq for 47 patients with OV.

Chr	Start	End	Score	# of patients	*P*-values	Genes
11	65642401	85427201	121563.30	21	0.00	ALG8
19	60001	8897001	−72426.36	40	0.03	
08	29425301	37553301	−61860.36	22	0.02	
19	12527301	19504701	47407.00	15	0.01	
05	43229501	46405601	46521.06	17	0.00	
03	160721001	178997301	45477.40	23	0.00	MECOM
10	85985501	91954501	−41721.05	17	0.02	PTEN
19	27731701	31299201	41154.68	19	0.05	CCNE1
04	52677801	61329601	40437.16	12	0.00	
15	89927001	95429901	37962.78	14	0.02	
18	18510801	24550601	37554.03	20	0.03	
14	20540801	23461401	37395.16	14	0.08	METTL17
08	124682001	137671301	35951.00	18	0.00	MYC
03	145312501	152115001	33691.04	15	0.02	
04	1743701	10725801	30664.76	20	0.02	TACC3
12	68439201	75601101	30556.17	7	0.03	
05	12795301	24076601	29573.97	23	0.00	
12	22892501	27931501	28443.16	13	0.05	KRAS
18	24550701	29219001	−28392.48	3	0.01	
03	110285601	116082001	27154.97	9	0.07	
01	38952601	41868301	26322.47	7	0.03	MYCL
03	115817701	117085401	−26257.95	9	0.05	
12	59901	7041901	25886.15	20	0.10	
14	65361901	75819301	−25236.16	26	0.00	
07	132300701	139244001	25116.75	9	0.06	
04	69261001	75913501	24782.67	8	0.09	ANKRD17
02	26158601	30486201	24553.85	8	0.02	
03	86256201	90504801	−24254.03	11	0.09	
04	49133401	49660001	−21703.25	17	0.10	
05	32915101	37315001	20138.94	8	0.07	
07	3488201	9166901	−16591.00	25	0.05	
16	10807101	18842701	15730.08	17	0.04	
14	90263501	94255601	−14860.60	4	0.10	
20	53261001	57941101	−14597.54	11	0.03	

Clusters are ordered by cluster scores. Among the genes in the clusters, some genes related to OV are specified.

**Table 4:** Comparison between GISTIC and WIFA-Seq for OV.

Chr	Genes	WIFA				GISTIC		
		cluster.start	cluster.end	cluster.length	FDC	peak.start	peak.end	peak.length
1	MYCL	38952601	41868301	2915701	1|2|3|4|5	39231800	41878565	2646766
1	MCL1	147794101	151448201	3654101	4|5			
3	MECOM	160721001	178997301	18276301	1|2|3|4|5			
4	TACC3	1743701	10725801	8982101	1|2|3|4|5			
4	ANKRD17	69261001	75913501	6652501	1|2|3|4|5			
5	TERT	9901	1850001	1840101	4|5			
8	SOX17	54155101	55670501	1515401	4|5			
8	MYC	124682001	137671301	12989301	1|2|3|4|5	126952807	132241106	5288300
10	PTEN	85985501	91954501	5969001	1|2|3|4|5	84746048	100217807	15471760
11	ALG8	65642401	85427201	19784801	1|2|3|4|5			
12	KRAS	22892501	27931501	5039001	1|2|3|4|5			
13	RB1	47517901	49138401	1620501	2|3|4|5	48833767	53194688	4360922
14	METTL17	20540801	23461401	2920601	1|2|3|4|5			
17	NF1	29302301	29948501	646201	2|3|4|5			
19	CCNE1	27731701	31299201	3567501	1|2|3|4|5	30258695	30343163	84469

For both methods, genes were included in clusters/peaks. Thus, start and end positions and the length of clusters/peaks are specified. A column FDC indicates FDCs that identified the given genes.

**Table 5:** Statistically significant clusters from WIFA-Seq for 28 patients with LUAD.

Chr	Start	End	Score	Patients	*P*-value	Gene
09	21028101	26141501	−19044.30	7	0.00	CDKN2A
05	26805201	30914501	18988.18	2	0.07	
08	113293301	118827401	−17228.32	4	0.04	
13	26627701	30384101	17073.60	2	0.00	*CDK8
19	29745001	36307301	14855.75	5	0.00	CCNE1
18	21903601	23631001	13671.30	2	0.04	*ZNF521
16	46385601	49388501	10241.17	4	0.06	
16	28991501	32062401	8451.53	4	0.08	
03	73849601	75930501	−6981.06	5	0.00	
17	19462601	22262901	−6924.59	13	0.09	
10	42354701	44696901	6398.16	6	0.04	
07	109434901	111629601	−5673.43	5	0.00	
04	49078801	49660001	−5172.21	8	0.00	
03	88609201	90504801	−5069.68	6	0.02	
05	131850601	134327701	−4491.72	3	0.10	
13	113517701	114976401	−4183.84	2	0.02	
15	48327301	53049801	−4101.93	3	0.00	
07	6633201	9254801	−4007.58	3	0.05	
21	22192401	23674201	3641.44	3	0.06	
10	55440801	57178101	−3532.84	3	0.01	
17	25265201	27720401	3511.43	3	0.04	
12	37880101	38436701	−2300.26	4	0.02	
02	89614801	89888601	−2257.03	4	0.01	

Clusters are ordered by the cluster scores. Among the genes in the clusters, some genes related to lung cancer are specified. Previously known lung cancer-related genes in the silver standard are shown without asterisks.

**Table 6:** Comparison between GISTIC and WIFA-Seq for LUAD.

Chr	Genes	WIFA				GISTIC		
		cluster.start	cluster.end	cluster.length	FDC	peak.start	peak.end	peak.length
5	TERT	784401	3276601	2492201	2|3|4|5			
9	PTPRD	7830301	9633501	1803201	4|5			
9	CDKN2A	21028101	26141501	5113401	1|2|3|4|5	21959052	21977193	18142
12	MDM2					68758042	70379446	1621405
19	CCNE1	29745001	36307301	6562301	1|2|3|4|5			

For both methods, the genes were included in clusters/peaks. Thus, start and end positions and the length of clusters/peaks are specified. A column FDC indicates FDCs that identified the given genes.
