# Corrigendum: Identification of cancer-driver genes in focal genomic alterations from whole genome sequencing data

**DOI:** 10.1038/srep32906

**Published:** 2016-09-14

**Authors:** Ho Jang, Youngmi Hur, Hyunju Lee

Scientific Reports 6: Article number: 2558210.1038/srep25582; published online: 05 09 2016; updated: 09 14 2016

This Article contains errors in the Acknowledgements section.

“This research was supported by Basic Science Research Program through the National Research Foundation of Korea (NRF) funded by the Ministry of Education (NRF-2013R1A1A2058053), and Institute for Information & communications Technology Promotion(IITP) grant funded by the Korea government(MSIP) (No. R-20150826-002098, Developing tools to detect cancer driver mutations using single-cell sequencing data). The funders had no role in study design, data collection and analysis, decision to publish, or preparation of the manuscript”.

should read:

“This research was supported by Basic Science Research Program through the National Research Foundation of Korea (NRF) funded by the Ministry of Education (NRF-2013R1A1A2058053), and the MSIP(The Ministry of Science, ICT and Future Planning), Korea and Microsoft Research, under ICT/SW Creative research program supervised by the IITP(Institute for Information & Communications Technology Promotion) (IITP-2015- R-20150826-002098)”.

