# Supplementary material for: Identification of cancer-driver genes in focal genomic alterations from whole genome sequencing data
Source: Sci Rep. 2016 May 9;6:25582. doi: 10.1038/srep25582 (PMC4860638; doi:10.1038/srep25582)
Supplement: Supplementary Information [file srep25582-s1.pdf]

Supplementary data: Identification of cancer-driver genes in focal genomic alterations from whole genome sequencing data

Ho Jang, Youngmi Hur and Hyunju Lee

Supplementary data list

Data	Descriptions	Pages
Table 1	Manually inspected copy number alteration status harboring known cancer genes from 37 GBM samples	Excel file
Table 2	Manually inspected copy number alteration status harboring known cancer genes from 47 OV samples	Excel file
Table 3	Manually inspected copy number alteration status harboring known cancer genes from 28 LUAD samples	Excel file
Table 4	Manually inspected copy number alteration status harboring known cancer genes from 70 LUAD samples	Excel file
Table 5	Statistically significant clusters identified by the WIFA algorithm with the parameters bin size = 100 base pairs, M = 10, C = 2.0, and d = 0.3 million base pairs and Ensembl gene annotations in the clusters for 37 GBM samples	Excel file
Table 6	The expression-level data for NPAS3 in 37 WGS GBM samples and CDK8 in 28 WGS lung adenocarcinoma samples. In the case of the 37 GBM samples, 29 expression-level data for 37 GBM samples exist	Excel file
Table 7	Comparison of the inspection length of silver standards confirmed by BIC-seq WGS segments in the 37 GBM samples	Excel file
Table 8	Ensembl gene annotations of GISTIC 2.0 peaks in the 37 GBM samples	Excel file
Table 9	Comparison of the inspection length of silver standards confirmed by BIC-seq WGS segments and TCGA SNP6 segments in 37 GBM samples	Excel file
Table 10	Statistically significant clusters identified by the WIFA algorithm with the parameters bin size = 100 base pairs, M = 10, C = 2.0, and d = 0.3 million base pairs and Ensembl gene annotations in the clusters for 47 OV samples	Excel file
Table 11	Ensembl gene annotations of GISTIC 2.0 peaks in the 47 OV samples	Excel file
Table 12	Comparison of the inspection length of silver standards confirmed by BIC-seq WGS segments and TCGA SNP6 segments in 47 OV samples	Excel file
Table 13	Statistically significant clusters identified by the WIFA algorithm with the parameters bin size = 100 base pairs, M = 10, C = 2.0, and d = 0.3 million base pairs and Ensembl gene annotations in the clusters for 28	Excel file

	lung adenocarcinoma samples	
Table 14	Ensembl gene annotations of GISTIC 2.0 peaks in the 28 lung adenocarcinoma samples	Excel file
Table 15	Comparison of the inspection length of silver standard genes confirmed by both BIC-seq WGS segments and TCGA SNP6 segments in 28 lung adenocarcinoma samples	Excel file
Table 16	Statistically significant clusters identified by the WIFA algorithm with the parameters bin size = 100 base pairs, M = 10, C = 2.0, and d = 0.3 million base pairs and Ensembl gene annotations in the clusters for 70 lung adenocarcinoma samples	Excel file
Table 17	Gene set enrichment test of GO biological process terms and inclusion status of COSMIC Cancer Gene Census genes	Excel file
Figure 1	The flowchart for generating y^{*}_{HIGH} for individual samples	4
Figure 2	The cost of silverstandard discovery in terms of gene inspection within WIFA-Seq clusters or GISTIC 2.0 alteration regions	5

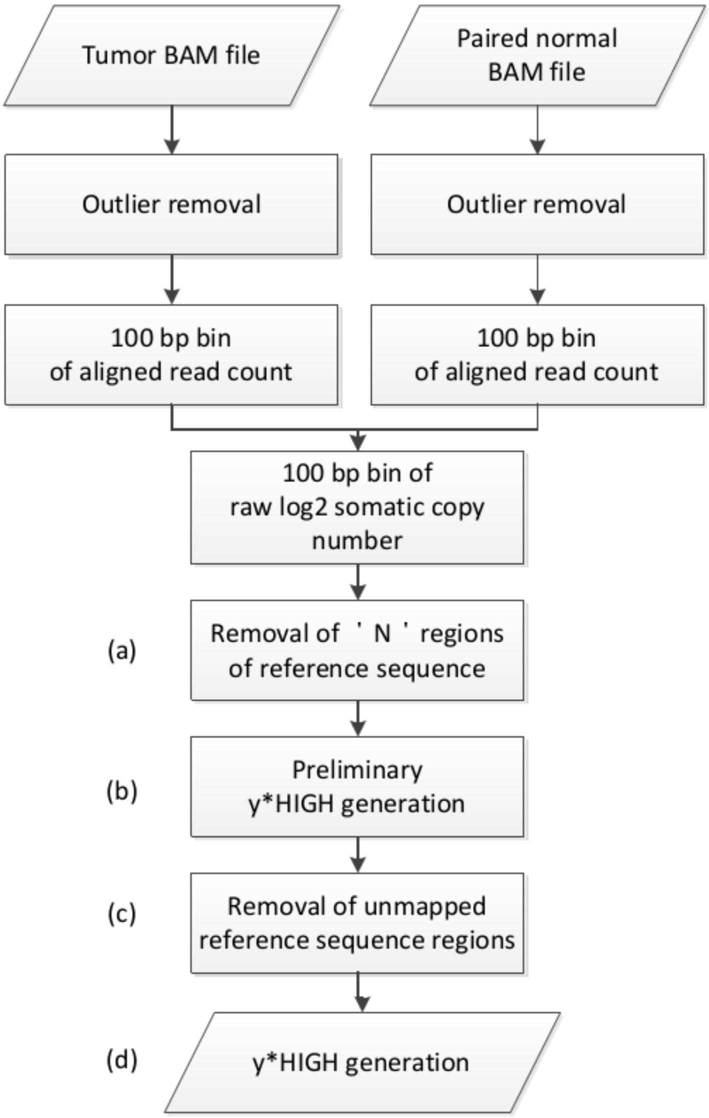

Supplementary Figure 1. The flowchart for generating y^*_{HIGH} for individual samples

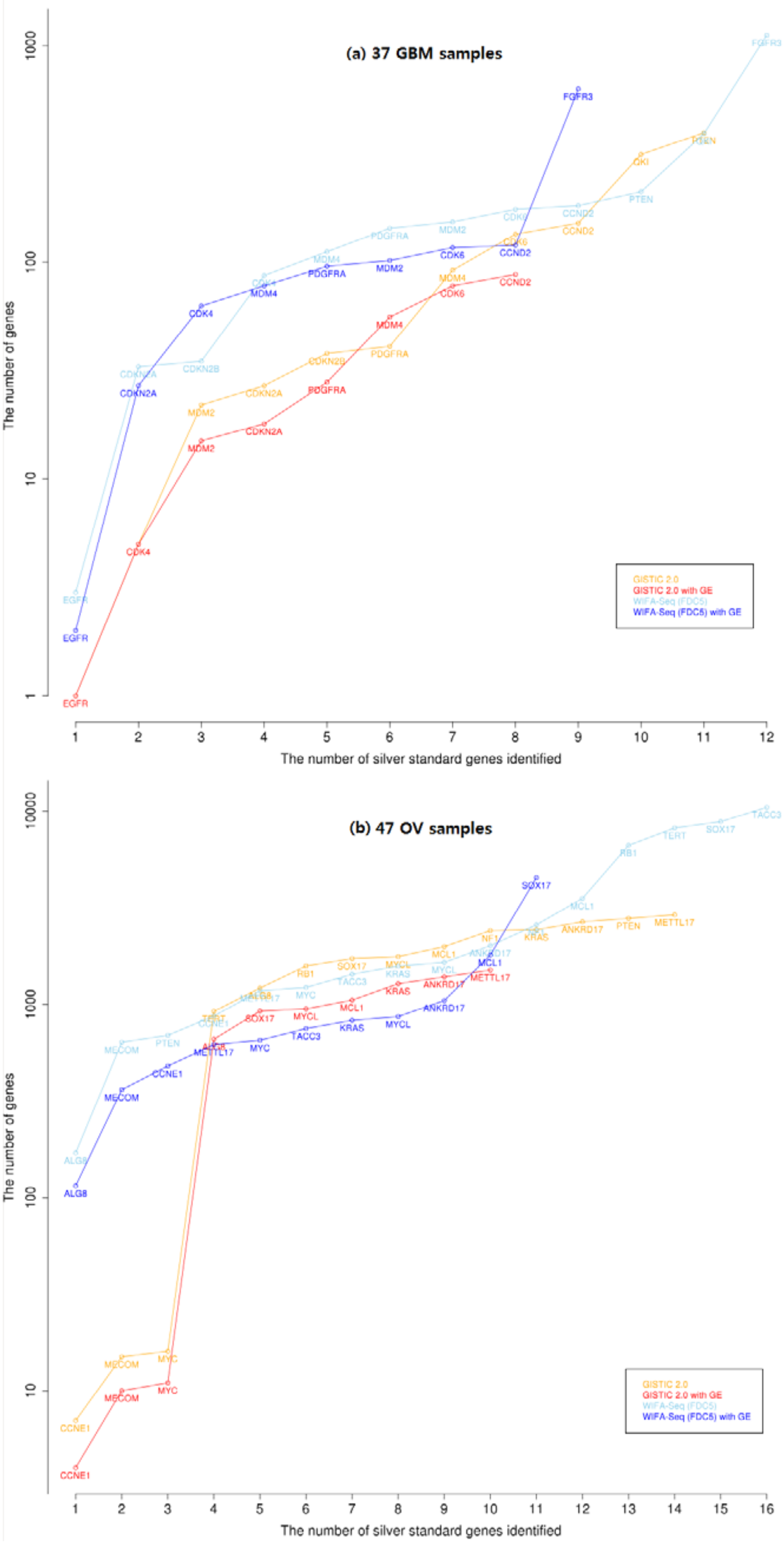

Supplementary Figure 2. The cost for identifying silver standard genes in terms of the number of genes required for inspection within WIFA-Seq clusters or GISTIC 2.0 alteration regions
